# Machine learning model for predicting rebleeding risk after endoscopic variceal ligation in esophageal variceal bleeding

**DOI:** 10.1038/s41598-026-58733-2

**Published:** 2026-06-19

**Authors:** Junyi Zhan, Keqiang Lu, Jinzhong Yu, Jie Chen, Feifei Xing, Dexin Wang, Shihao Zhang, Mingyan Yang, Ping Liu, Jian Wang, Yongping Mu

**Affiliations:** 1 Cell Biology Laboratory, Shuguang Hospital Affiliated to Shanghai University of Traditional Chinese Medicine (TCM), 528, Zhangheng Road, Pudong District, Shanghai, 201203 China; 2 Institute of Liver Diseases, Shanghai Academy of Chinese Medicine, Shanghai, China; 3 Clinical Key Laboratory of TCM of Shanghai, Shanghai, China; 4 Key laboratory of Liver and Kidney Disease of the Ministry of Education, Shanghai, China; 5 Department of Gastrointestinal Endoscopy, Shuguang Hospital Affiliated to Shanghai University of TCM, Shanghai, China; 6 Department of Gastroenterology and Hepatology, Zhongshan Hospital Affiliated to Fudan University, 180, Fenglin Road, Xuhui District, Shanghai, 200032 China; 7 Shanghai Institute of Liver Disease, Shanghai, China; 8 Evidence-Based Medicine Center, Fudan University, Shanghai, China; 9 Department of Gastroenterology, Hangzhou Hospital of TCM, Hangzhou, China

**Keywords:** Esophageal variceal bleeding, Rebleeding, Endoscopic variceal ligation, Prediction model, Machine learning, Gastroenterology, Risk factors

## Abstract

Rebleeding is a severe complication following recovery from esophageal variceal bleeding (EVB), yet robust predictive tools for assessing post-treatment risk after endoscopic variceal ligation (EVL) therapy remain scarce. This study developed and independently validated a machine learning (ML) model using multidimensional clinical data to predict 1-year rebleeding risk. Two independent cohorts were included: a retrospective cohort (*n* = 373) for model development and a prospective cohort (*n* = 119) for validation, with a one-year rebleeding endpoint. Predictors were identified using Recursive Feature Elimination (RFE), and eight ML algorithms were evaluated. Each algorithm was optimized via 5-fold cross-validation. The model with optimal performance was chosen to develop an online computational platform. RFE identified eight key predictors. The XGBoost model demonstrated superior predictive performance in both the training and validation cohorts, achieving AUCs of 0.883 and 0.887, respectively. This model was subsequently implemented in an online clinical platform for individualized 1-year rebleeding risk assessment. Our findings establish XGBoost as an effective tool for predicting EVB rebleeding risk, providing an evidence-based decision aid for post-EVL management.

**Supplementary Information:**

The online version contains supplementary material available at https://doi.org/10.1038/s41598-026-58733-2.

## Introduction

Esophageal variceal bleeding (EVB) is a severe clinical event resulting from portal hypertension in liver cirrhosis patients, with a first-time bleeding mortality rate of 12% to 22%^1,2^. Rebleeding is a frequent and serious complication following recovery from EVB. In the absence of effective secondary prevention, the rebleeding rate can reach up to 60%, with a mortality risk of approximately 33%, significantly increasing the disease burden^3,4^. Endoscopic variceal ligation (EVL) is currently widely used for acute heamostasis and secondary prevention in EVB patients^1^. However, post-EVL rebleeding rates remain high at 22% to 33%, underscoring the need for improved individualized risk management^5^. Developing a rebleeding risk prediction model based on individual patient risk factors could enhance disease management and clinical outcomes.

Previous studies have identified key aspects related to rebleeding risk in EVB patients, including demographic characteristics, laboratory parameters, comorbidities and complications, endoscopic findings, radiological features and medication effects^6^. However, most existing models rely heavily on laboratory parameters, lacking a comprehensive assessment of multidimensional risk factors, and are seldom independently validated, limiting their generalizability^7–9^. In contrast, machine learning (ML) offers an advanced approach to data analysis, enabling the integration of complex, multidimensional data to uncover hidden predictive factors and improve predictive accuracy^10^.

The present study aimed to integrate multidimensional clinical features in EVB patients to evaluate the predictive performance of various ML models for 1-year rebleeding risk and to perform independent validation. Ultimately, we developed a rebleeding risk prediction platform based on the best-performing ML model, aiming to support clinical application and provide a scientific basis for individualized treatment and follow-up.

## Method

### Study population

The training cohort comprised two retrospective cohorts totaling 373 cirrhotic patients with EVB treated by EVL. Of these, 201 patients were enrolled at Shuguang Hospital Affiliated with Shanghai University of Traditional Chinese Medicine, and 172 at Zhongshan Hospital Affiliated with Fudan University (March 2021-February 2023). Validation used prospective cohorts of 119 EVB patients undergoing EVL between March-November 2023 (57 from Shuguang Hospital; 62 from Zhongshan Hospital). All patients completed one-year follow-up via telephone interviews, outpatient visits, and medical record reviews.

This study was conducted in accordance with the Transparent Reporting of a Multivariable Prediction Model for Individual Prognosis or Diagnosis (TRIPOD) guidelines^11^. The study adhered to the principles of the Declaration of Helsinki and received ethical approval from the Ethics Committees of Shuguang Hospital (approval number: 2017-560-43) and Zhongshan Hospital (approval number: B2023-055R). Given that the retrospective cohort data did not contain any personal identifiers and were anonymized before use, the need for informed consent was waived. For the prospective cohort, written informed consent was obtained from all participants or their legal guardians.

### Eligibility criteria

Inclusion Criteria: (1) Patients diagnosed with cirrhosis (based on clinical characteristics, laboratory and imaging tests, or liver biopsy); (2) Patients admitted due to endoscopically confirmed EVB; (3) Age ≥ 18 years; (4) Patients undergoing EVL for the first time.

Exclusion Criteria: (1) Patients with prior EVL or tissue adhesive/sclerotherapy injection; (2) Patients who had previously undergone transjugular intrahepatic portosystemic shunt (TIPS), balloon-occluded retrograde transvenous obliteration, splenectomy, or liver transplantation; (3) Patients diagnosed with hepatocellular carcinoma or other malignant tumors at the time of recruitment; (4) Patients with severe cardiac or pulmonary diseases, or severe renal insufficiency; (5)Patients with a follow-up period of less than one year.

### Therapeutic interventions

All patients in both the training and validation cohorts received uniform management in accordance with Baveno VII^1^ and AASLD guidelines^12,13^. Initial care included vasoactive drugs and prophylactic antibiotics within 12 h, followed by prompt endoscopic evaluation, with hemostasis performed as necessary. Secondary prophylaxis combined EVL with non-selective beta-blocker (NSBB), adjusted per institutional protocols and clinician judgment.

Each center had two senior endoscopists with over five years of therapeutic experience responsible for performing endoscopic procedures. EVL was conducted using multiband ligation from the cardia to proximal esophagus. Concurrent gastric varices received lauroylmorpholine and tissue adhesive injections via therapeutic endoscopy.

### Outcomes

The primary outcome was one-year rebleeding, defined as recurrent hematemesis, melena, hemodynamic instability (systolic blood pressure drop > 20 mmHg or heart rate increase > 20 bpm), or hemoglobin decline > 30 g/L post-initial control^14^. Since rebleeding within the first 5 days defines treatment failure, the observation for the primary outcome commenced on day 6^1,14^. Events were clinically identified and subsequently confirmed by repeat endoscopy performed by the site endoscopy specialists.

### Candidate predictors and data collection

Baseline admission variables were analyzed across six domains: demographics, laboratory parameters, comorbidities and complications, endoscopic findings^15^, radiological features, and medications. Bleeding signs included active spurting/oozing or red/white plugs during endoscopy^15,16^. The primary outcome of rebleeding was prospectively collected by dedicated data collectors during follow-up. Two data managers independently verified clinical records.

### Statistical analysis

All statistical analyses were performed using R version 4.3.2 (https://www.R-project.org). Descriptive statistics are expressed as *N* (%), $\bar {x}$± SD, or median (Q1, Q3), as appropriate. Missing data were handled using multiple imputation, and variables with over 15% missing values were excluded from analysis. Comparisons of continuous variables with normal distribution between groups were performed using Student’s t-test, while the Mann-Whitney U test was applied for non-normally distributed continuous variables. Categorical variables were compared using Pearson’s chi-square test or Fisher’s exact test. A *P* < 0.05 (two-tailed) was considered indicative of statistical significance.

Feature selection employed Recursive Feature Elimination (RFE) to iteratively identify optimal predictors while minimizing overfitting in high-dimensional data^17,18^. Eight ML algorithms were implemented: decision tree (DT), random forest (RF), k-nearest neighbors (KNN), logistic regression (LR), XGBoost, multilayer perceptron (MLP), support vector machine (SVM), and LightGBM.

Model development utilized five-fold cross-validation for hyperparameter tuning and validation, with subsequent independent validation performed on prospective cohorts. Performance evaluation included area under the receiver operating characteristic curve (AUC), accuracy, F1-score, sensitivity, and specificity. The top-performing model was selected based on robustness across validation phases. Clinical net benefit was evaluated using Decision Curve Analysis (DCA).

Subgroup analyses evaluated predictive consistency across age (< 60, ≥ 60), sex (male, female), cirrhosis etiology (hepatitis B virus infection, autoimmune liver disease, alcohol-related, and other), variceal type (esophageal varices, combined gastric varices), use of emergency endoscopic hemostasis (yes, no), and NSBB usage (yes, no).

The interpretability of the final predictive model was enhanced using Shapley Additive Explanations (SHAP) to determine the contribution of each feature to the prediction outcome. To support real-world clinical application, the model was integrated into an online platform based on the Shiny R package to facilitate clinical accessibility and use.

## Result

### Comparison of clinical characteristics

**Fig. 1:**
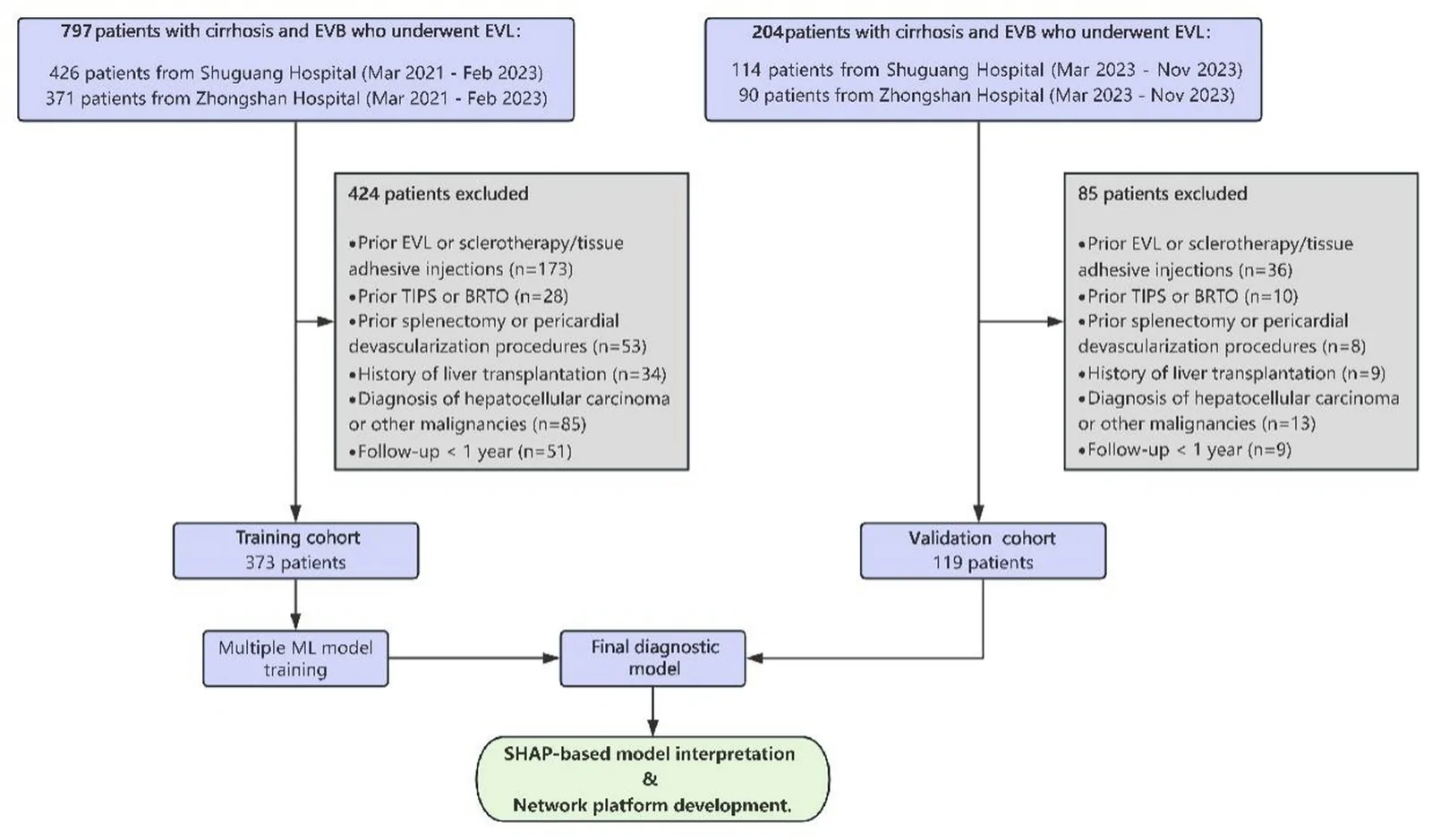
Study flow chart.

As illustrated in Fig. 1, the initial training cohort comprised 797 patients with cirrhosis and EVB, of whom 424 were excluded due to not meeting the inclusion criteria, resulting in a final sample of 373 patients. The validation cohort included 204 patients, with 119 meeting eligibility criteria and included in the study. In total, 492 patients with cirrhosis and EVB were enrolled. Baseline characteristics and missing data are detailed in Table 1. After excluding variables with more than 15% missing data, 54 variables were retained for statistical analysis to ensure accuracy. No significant difference was found in rebleeding rates between the training and validation cohorts (22.79% vs. 24.37%, *P* = 0.82), with most clinical characteristics being similar. However, thrombin time was significantly longer in the training cohort compared to the validation cohort (18.05 [17–19.17] vs. 16.6 [15.9–17.65], *P* < 0.01). Supplementary Table 1 provides a comparison of baseline characteristics between patients with and without rebleeding in the cirrhosis and EVB groups.

**Table 1:** Baseline characteristics and outcomes of the training and validation cohorts.

Characteristic	Training cohort(*n* = 373)	Validation cohort(*n* = 119)	*P*-Value
Demographics characteristics			
Age (yr)	58.05 ± 12.21	60.04 ± 12.62	0.13
Sex, n (%)			0.8
Female	148 (39.68)	45 (37.82)	
Male	225 (60.32)	74 (62.18)	
Smoking history, n (%)	68 (18.28)	15 (12.71)	0.21
Drinking history, n (%)	87 (23.39)	25 (21.19)	0.71
Surroundings, n (%)			0.09
Rural	106 (28.42)	44 (36.97)	
Suburban	74 (19.84)	15 (12.61)	
Urban	193 (51.74)	60 (50.42)	
Symptom, n (%)			0.1
Melena or hematochezia	121 (32.44)	49 (41.18)	
Hematemesis	252 (67.56)	70 (58.82)	
Etiology, n (%)			0.52
Chronic HBV infection	173 (46.38)	53 (44.54)	
Autoimmune liver disease	70 (18.77)	20 (16.81)	
Alcohol-related	59 (15.82)	16 (13.45)	
Other	71 (19.03)	30 (25.21)	
Course of cirrhosis (year)	2 (0.5–6)	2.5 (0.5–6)	0.7
Family history of cirrhosis, n (%)	46 (12.37)	12 (10.08)	0.61
Endoscopic findings			
Received emergency endoscopic hemostasis, n (%)	36 (9.65)	10 (8.40)	0.82
Types of varices, n (%)			0.07
EV	166 (44.50)	41 (34.45)	
EV combined with GV	207 (55.50)	78 (65.55)	
Red color sign, n (%)	353 (94.64)	113 (94.96)	1
Bleeding signs, n (%)	64 (17.16)	19 (15.97)	0.87
Form of varices, n (%)			1
F1	64 (17.16)	21 (17.65)	
F2-F3	309 (82.84)	98 (82.35)	
Location of varices, n (%)			0.79
Low to middle	313 (84.14)	102 (85.71)	
High	59 (15.86)	17 (14.29)	
Numbers of varices	4 (4–4)	4 (4–4)	0.08
Diameter of varices (cm)	0.85 (0.8–1)	1 (0.8–1)	0.25
Number of EVL (times)	2 (1–3)	2 (1–3)	0.59
Received sclerotherapy injections, n (%)	162 (43.43)	63 (52.94)	0.09
Received tissue glue injections, n (%)	146 (39.14)	58 (48.74)	0.08
Treatment failure, n (%)	3 (0.80)	1 (0.84)	1
Comorbidities and complications			
Ascites, n (%)			0.61
None	131 (35.12)	38 (31.93)	
Mild	144 (38.61)	52 (43.70)	
Moderate and severe	98 (26.27)	29 (24.37)	
Encephalopathy, n (%)	13 (3.49)	3 (2.52)	0.83
Diabetes, n (%)	79 (21.24)	23 (19.33)	0.75
Hypertension, n (%)	76 (20.43)	28 (23.53)	0.55
Hemorrhagic shock, n (%)	11 (2.96)	0 (0.00)	0.12
Heart disease, n (%)	16 (4.30)	9 (7.56)	0.24
Laboratory parameters			
Total bilirubin (µmol/L)	19.1 (13.5–30.2)	17.45 (12.83–25.98)	0.11
Alanine aminotransferase (U/L)	24 (16–35)	23 (17–33)	0.64
Aspartate aminotransferase (U/L)	32 (23.25–46)	31.5 (25–44.75)	0.79
Alkaline phosphatase (U/L)	86.5 (65.25–115.5)	84 (67.25–116.5)	0.98
Albumin (g/L)	32.76 ± 6.18	33.9 ± 6.06	0.08
White blood cell (×10^9^/L)	2.96 (2.03–4.64)	2.9 (2.16–4.49)	0.84
Red blood cell (×10^9^/L)	3.08 ± 0.74	3.15 ± 0.82	0.40
Hemoglobin (g/L)	88.23 ± 23.48	88.52 ± 25.49	0.91
Platelet count (×10^9^/L)	66 (46–90.25)	70 (46–99.5)	0.66
Prothrombin time (s)	14.7 (13.65–16.1)	14.3 (13.55–15.45)	0.10
INR (unit)	1.27 (1.16–1.39)	1.24 (1.17–1.33)	0.14
D-dipolymer (ug/ml)	1.37 (0.54–3.11)	1.38 (0.62–3.41)	0.55
Thrombin time (s)	18.05 (17–19.17)	16.6 (15.9–17.65)	< 0.01
Fibrinogen (g/L)	192.89 ± 78.98	207.08 ± 69.8	0.08
CTP class, n (%)			0.14
A (5–6)	141 (37.80)	54 (45.38)	
B (7–9)	204 (54.69)	61 (51.26)	
C (10–13)	28 (7.51)	4 (3.36)	
Radiological features			
Portal vein thrombosis, n (%)	87 (23.58)	36 (30.25)	0.18
Splenic vein diameter (mm)	9 (7.05–11)	10 (7.97–12)	0.07
Inner diameter of portal vein trunk (mm)	12.7 (11–14)	13 (12–14)	0.06
Portal vein flow velocity (cm/s)	20 (16–24)	18.2 (14–23)	0.07
Medication usage			
Use of NSBB, n (%)	246 (65.95)	82 (68.91)	0.63
Use of PPI, n (%)	350 (93.83)	114 (95.80)	0.56
Use of antibiotics, n (%)	255 (68.36)	89 (74.79)	0.22
Outcomes			
Rebleeding within 1y, n (%)	85 (22.79)	29 (24.37)	0.82

Abbreviations: EV: esophageal varices; GV: gastric varices; EVL: endoscopic variceal ligation; INR: international normalized ratio; CTP class: Child-Turcotte-Pugh class; NSBB: non-selective beta-blockers; PPI: proton pump inhibitor.

During the 1-year follow-up, 11 patients developed hepatocellular carcinoma. Prior to rebleeding, one patient received TIPS, and another underwent splenectomy. A total of 12 patients died, with 11 deaths attributed to hemorrhagic shock or multiorgan failure related to EVB and one death due to sepsis.

### Selection of key predictors

Recursive feature elimination (RFE) based on a random forest algorithm was applied to screen 54 independent variables in the training cohort. The feature selection process yielded an optimal subset of eight clinical predictors when the model achieved peak predictive accuracy (81.74%) (as shown in Supplementary Fig. 1). These predictors included concentrations of albumin (ALB), alanine aminotransferase (ALT), aspartate aminotransferase (AST), and hemoglobin (HB), as well as the presence of ascites, portal vein thrombosis (PVT), and bleeding signs, along with portal vein flow velocity (Pvv).

### Performance comparison of different ML models

**Fig. 2:**
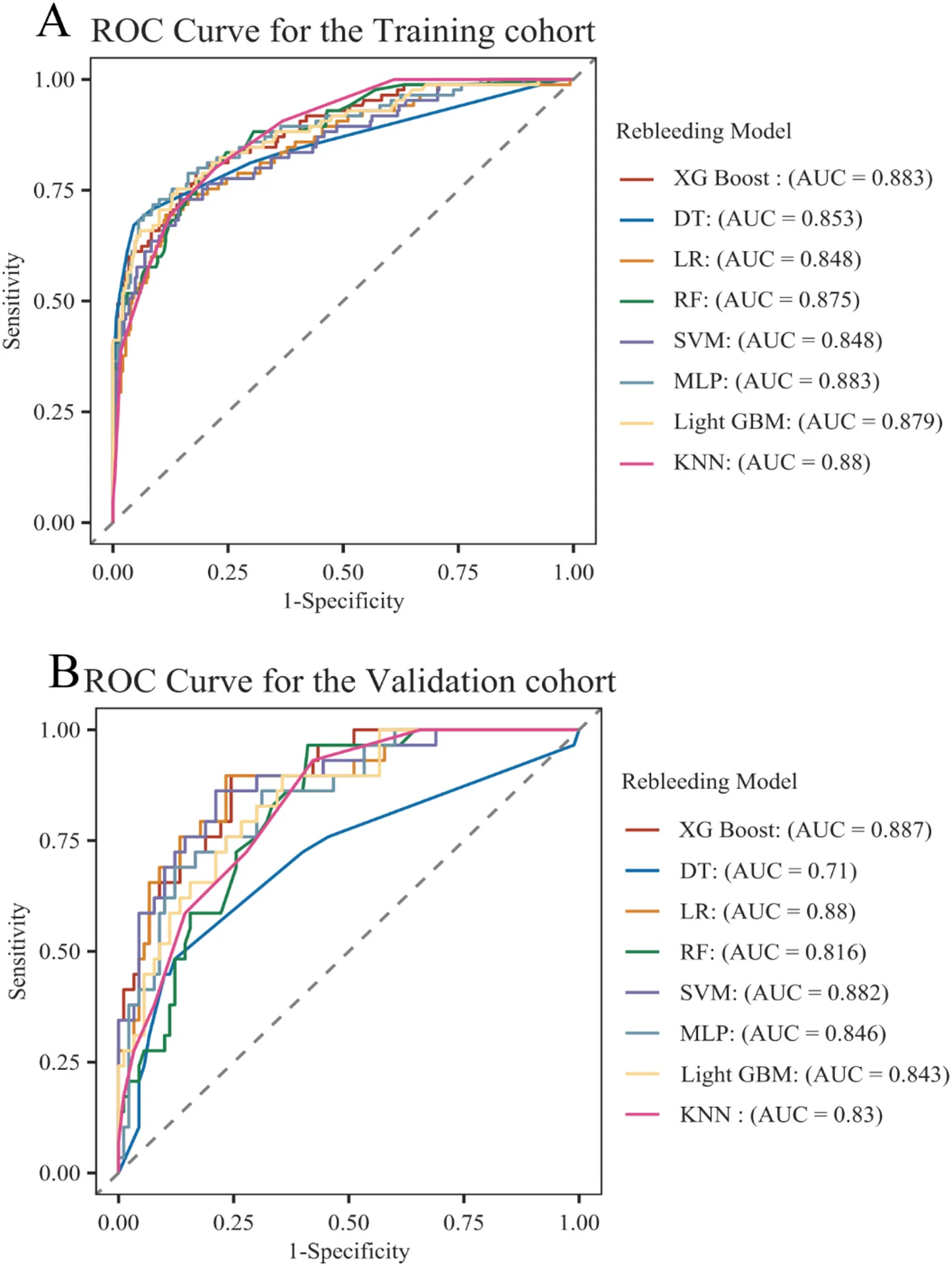
AUC comparison of eight machine learning models in predicting EVB rebleeding in (**A**) the Training cohort and (**B**) the Validation cohort. Abbreviations: DT: decision tree; KNN: k-nearest neighbors; LightGBM: light gradient boosting machine; LR: Logistic regression; MLP: multilayer perceptron; RF: random forest; SVM: support vector machine; XGBoost: extreme gradient boosting.

**Fig. 3:**
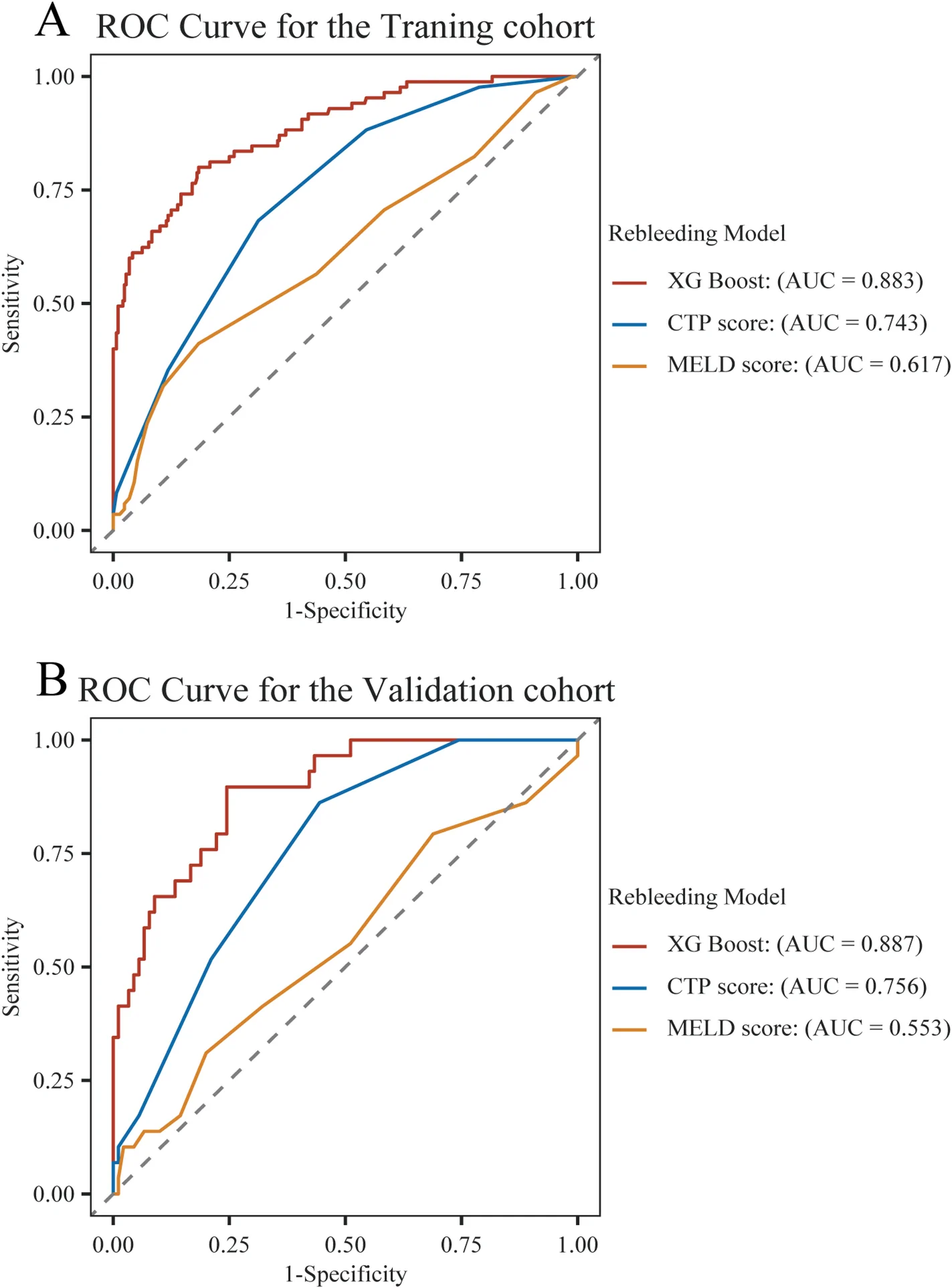
AUC comparison between the XGBoost model and conventional liver disease risk scores for EVB rebleeding in (**A**) the Training cohort and (**B**) the Validation cohort. Abbreviations: XGBoost: extreme gradient boosting; CTP: Child-Turcotte-Pugh; MELD: Model for End-stage Liver Disease.

Based on the selected predictors, eight ML models (DT, RF, KNN, LR, XGBoost, MLP, SVM, and LightGBM) were trained and evaluated. In the training cohort, all models demonstrated robust predictive performance, with AUC exceeding 0.80. The XGBoost algorithm achieved the highest discriminative ability (AUROC = 0.883, 95% CI: 0.841–0.925). Following five-fold cross-validation, the mean AUROC values ranged from 0.70 to 0.85 across models, with XGBoost maintaining superior performance (AUROC = 0.821 ± 0.056). Independent validation confirmed generalizability, yielding AUROC ranges of 0.70–0.90, where XGBoost again exhibited optimal predictive accuracy (AUROC = 0.887, 95% CI: 0.824–0.951) (Fig. 2). Notably, XGBoost significantly outperformed Child-Turcotte-Pugh and Model for End-Stage Liver Disease scores in both cohorts (Fig. 3). Detailed performance metrics for each model are listed in Table 2. Based on these results, XGBoost was selected as the final risk prediction model.

**Table 2:** Predictive performance of eight machine learning models.

	AUC (%)	Accuracy (%)	Sensitivity (%)	Specificity (%)	F1 score (%)
Training cohort					
DT	85.35	89.01	95.49	67.06	93.06
LR	84.80	84.18	88.54	69.41	89.63
RF	87.45	80.43	81.25	77.65	86.51
XGBoost	88.29	81.23	81.60	80.00	87.04
SVM	84.84	82.31	85.07	72.94	88.13
MLP	88.28	86.86	91.32	71.76	91.48
Light GBM	87.87	83.65	86.11	75.29	89.05
KNN	88.04	78.28	77.78	80.00	84.69
Cross-validation					
DT	70.53	76.93	89.41	33.31	85.52
LR	81.24	83.65	94.46	46.29	89.88
RF	78.62	79.08	98.60	14.26	87.83
XGBoost	82.08	82.31	95.49	37.99	89.19
SVM	81.53	81.49	94.89	37.56	88.74
MLP	79.01	80.96	95.82	32.12	88.47
Light GBM	79.01	80.96	95.82	32.12	88.47
KNN	78.68	80.97	96.94	27.65	88.72
Validation cohort					
DT	71.02	78.99	90.00	44.83	86.63
LR	87.97	84.87	90.00	68.97	90.00
RF	81.55	77.31	83.33	58.62	84.75
XGBoost	88.74	78.99	81.11	72.41	85.38
SVM	88.20	83.19	86.67	72.41	88.64
MLP	84.60	82.35	87.78	65.52	88.27
Light GBM	84.25	78.99	83.33	65.52	85.71
KNN	82.97	72.27	72.22	72.41	79.75

Abbreviations: AUC: area under the receiver operating characteristic curve; DT: decision tree; LR: logistic regression; RF: random forest; XGBoost: extreme gradient boosting; SVM: support vector machine; MLP: multilayer perceptron; Light GBM: light gradient boosting machine; KNN: k-nearest neighbors.

Supplementary Fig. 2 illustrates the calibration performance of the eight ML models in both the training and validation cohorts. The XGBoost model demonstrated good fit in both cohorts, indicating a strong agreement between predicted and observed values.

### Evaluation of XGBoost model performance using DCA curves

DCA revealed that the XGBoost model provided substantial clinical net benefit across low-to-moderate threshold probabilities. The net benefit progressively diminished with increasing threshold values, indicating reduced clinical utility at higher decision thresholds (Supplementary Fig. 3). DCA revealed maximal clinical utility at low-to-moderate probability thresholds, supporting the prioritization of high-sensitivity diagnostic settings where missed true positives incur critical consequences.

### Subgroup analysis

To evaluate the diagnostic performance of the XGBoost model across patient subgroups, the two cohorts were combined, and patients were stratified by age, sex, cirrhosis etiology, variceal type, use of emergency endoscopic hemostasis, and NSBB usage. Subgroup analysis showed that the XGBoost model consistently demonstrated great predictive performance across all subgroups (Table 3).

**Table 3:** XGBoost Model performance in subgroup analyses.

	AUC (%)	Accuracy (%)	Sensitivity (%)	Specificity (%)	F1 score (%)
Age					
< 60	87.64	80.97	82.23	76.00	87.33
≥ 60	88.93	80.41	80.66	79.69	85.88
Sex					
Male	88.09	81.27	81.12	81.82	87.10
Female	89.12	79.79	82.07	72.92	85.92
Etiology of Cirrhosis					
Chronic HBV infection	88.54	81.42	81.71	80.39	87.20
Autoimmune liver disease	89.72	83.33	85.51	76.19	88.72
Alcohol-related	88.58	80.00	78.57	84.21	85.44
Other	85.79	77.23	79.49	69.57	84.35
Types of varices					
EV	90.70	82.49	81.71	84.91	87.58
EV combined with GV	86.57	79.27	81.31	72.13	85.93
Emergency endoscopic hemostasis					
Yes	93.81	82.35	73.33	95.24	83.02
No	87.01	80.50	82.18	74.19	86.93
Use of NSBB					
NSBB Group	87.85	78.35	79.07	75.71	85.18
Non-NSBB Group	89.72	85.37	86.67	81.82	89.66

Abbreviations: AUC: area under the receiver operating characteristic curve; HBV: hepatitis B virus; EV: esophageal varices; GV: gastric varices; NSBB: non-selective beta-blockers.

### Model interpretation and application

**Fig. 4:**
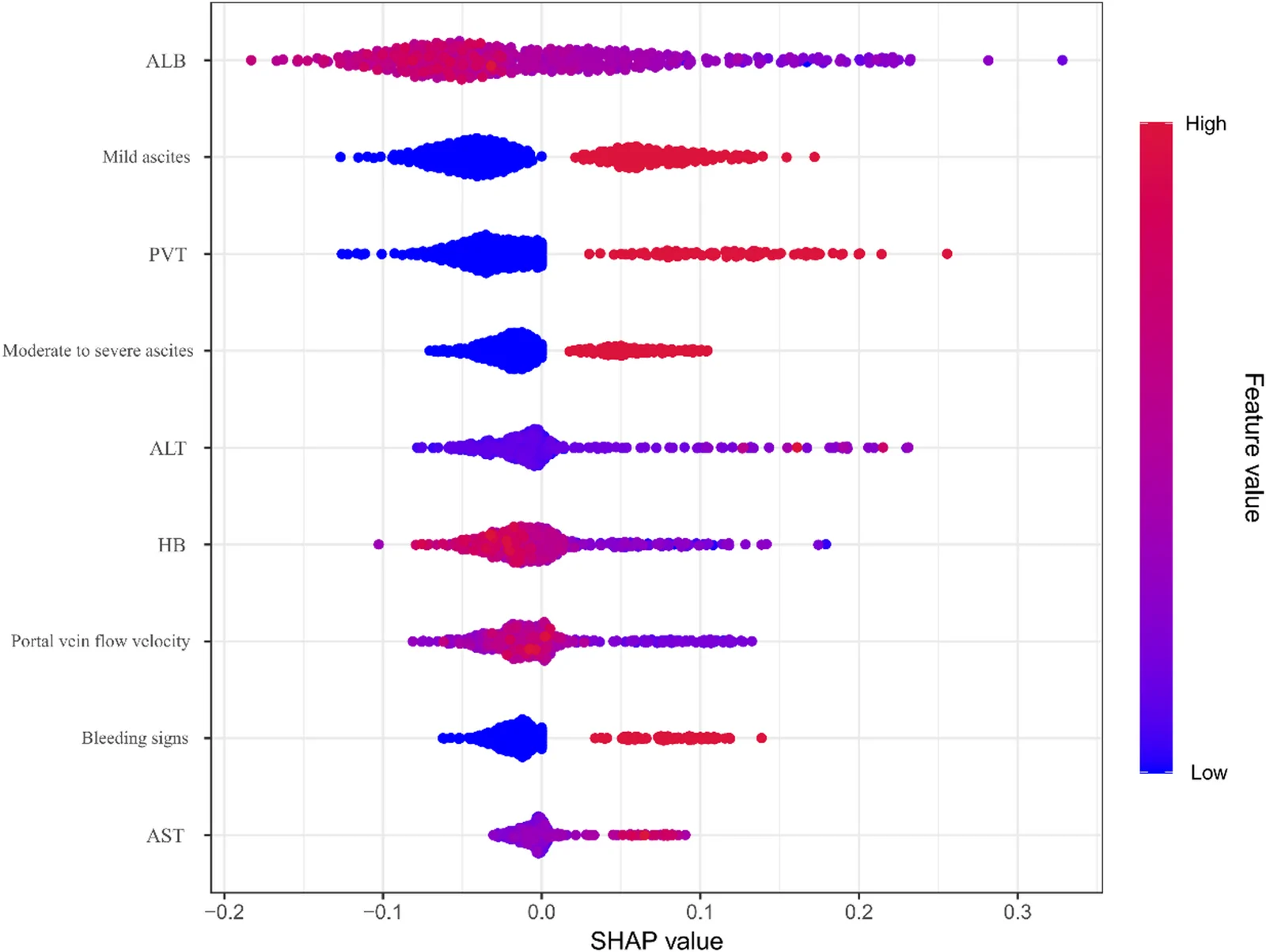
SHAP analyses of the XGBoost model for predicting EVB rebleeding.

SHAP plots were used to visually interpret the impact of predictors on model predictions. Specifically, SHAP value magnitude (indicated by color) and the distribution of each predictor along the horizontal axis (representing rebleeding probability) illustrated each predictor’s influence on outcomes. For concentrations of ALT, AST, and the presence of ascites, PVT, and bleeding signs, higher SHAP values (indicated in red) corresponded with increased rebleeding risk, identifying these predictors as risk factors. In contrast, higher ALB and HB levels, along with Pvv (indicated in blue) were associated with lower rebleeding risk, identifying it as a protective factor against rebleeding (Fig. 4). Supplementary Fig. 4 illustrates the SHAP dependence plots for the eight clinical predictors, demonstrating the impact of each feature on the model’s rebleeding risk predictions.

The XGBoost model, built upon these eight predictors, was subsequently operationalized into an online risk-calculation tool (https://p2awsp-0-0.shinyapps.io/1Year_EV_Rebleeding_model/) to facilitate personalized clinical decision-making for cirrhotic EVB patients.

## Discussion

Rebleeding is a common and serious complication in cirrhotic patients with EVB. Although EVL achieves a hemostasis rate of over 90%, it does not address the underlying issue of portal hypertension, resulting in frequent rebleeding episodes^1,19^. EVB further exacerbates liver dysfunction and is often accompanied by complications such as hypersplenism and infection^20^. With portal hypertension difficult to control, mortality can reach up to 33%^2^. Due to the complex pathophysiology and multiple clinical factors involved in rebleeding, precise risk prediction tools are currently lacking^1^. Therefore, developing a systematic rebleeding risk prediction platform to support individualized follow-up and treatment decisions is crucial for reducing rebleeding risk and improving patient outcomes.

In this longitudinal cohort study, we screened 54 clinical features and ultimately developed and validated a rebleeding prediction model, which is based on eight key variables including concentrations of ALT, AST, ALB, and HB, the presence of ascites, PVT, and bleeding signs, along with Pvv. The XGBoost model demonstrated excellent performance in predicting the 1-year rebleeding risk in EVB patients, surpassing seven other modeling methods, with an AUROC of 0.883 in the training cohort and 0.887 in validation cohort. The results of DCA curves indicate that the XGBoost model is well-suited for decision-making scenarios that prioritize high sensitivity. Thus, its application in the diagnosis of rebleeding events can maximize the identification of cases at risk for rebleeding and help prevent its occurrence. Moreover, the XGBoost model exhibited outstanding predictive performance across various subgroups, including age, sex, cirrhosis etiology, variceal type, use of emergency endoscopic hemostasis, and NSBB usage, demonstrating its broad applicability across different clinical contexts. Additionally, the model’s predictors are readily accessible in clinical practice, allowing for rapid risk assessment of rebleeding and thereby providing valuable support for adjusting follow-up strategies.

Previous studies on EVB rebleeding risk have identified various potential influencing factors, encompassing demographic characteristics, laboratory parameters, comorbidities and complications, endoscopic findings, radiological features, and medication effects^6,21–23^. Based on these factors, several clinical strategies for rebleeding prediction have been developed. In a study by Swarna et al.^24^, the platelet-albumin-bilirubin score was developed as a risk stratification tool to predict 90-day rebleeding outcomes in cirrhotic patients with acute variceal bleeding, achieving an AUROC of 0.803. The study also reported AUROC values of 0.732 and 0.71 for the Child-Turcotte-Pugh and MELD-Na scores, respectively. However, these approaches often fall short of the high accuracy required in clinical practice and have limited ability to predict long-term rebleeding outcomes. ML provides advantages in handling large datasets and identifying potential risk factors, significantly enhancing predictive accuracy. In 2023, Liu et al.^25^ developed a rebleeding prediction model using the XGBoost algorithm, incorporating 15 variables such as liver stiffness measurement, platelet count, body mass index, blood urea nitrogen, and cholesterol levels. The model achieved an AUROC of 0.931 in the training set for predicting 1-year rebleeding risk and 0.767 in internal validation. Nevertheless, this model has several limitations. First, it requires numerous variables, which adds complexity to its clinical application. Second, it does not fully account for key risk factors, including comorbidities and endoscopic findings. Additionally, the model lacks independent validation, limiting its generalizability, and has not been integrated into a clinical application platform, reducing its practical utility.

In this study, the predictors included in the XGBoost model reflected various characteristics of patients with liver cirrhosis and EVB, covering liver and kidney dysfunction, severity of portal hypertension, comorbidities and complications, and variceal characteristics. Low serum ALB and HB levels and elevated ALT and AST levels were identified as risk factors for EVB rebleeding. Reduced ALB levels indicate impaired liver synthetic function, while elevated ALT and AST primarily reflect hepatocellular injury. Together, these parameters signify the extent of liver dysfunction and are closely associated with cirrhosis progression^26,27^. Notably, HB levels directly reflect the severity of acute blood loss in EVB, with lower values correlating with compromised oxygen delivery and hemodynamic instability, which may exacerbate portal hypertensive pathophysiology^28^. The presence of ascites was also identified as a key risk factor for rebleeding, as it indicates elevated portal pressure and exacerbates portal hypertension through increased intra-abdominal pressure. Furthermore, patients with ascites often experience bacterial translocation, potentially leading to inflammatory responses and endothelial dysfunction, thereby increasing the risk of rebleeding^29^. All diagnoses of PVT in this study were confirmed by computed tomography. PVT has been established as a risk factor for rebleeding, likely due to increased portal pressure from thrombotic obstruction, altered hemodynamics, and increased flow and pressure within collateral circulation^30^. Importantly, reduced main Pvv—a hemodynamic consequence of elevated intrahepatic vascular resistance—served as an independent predictor of rebleeding in our cohort. This velocity reduction reflects progressive portal pressure elevation and correlates with compensatory collateral vessel dilation, which predisposes to variceal rupture^31^. Characteristics related to endoscopic examination and treatment have often been overlooked in previous studies. However, our findings indicate that endoscopic bleeding signs are important risk factors for EVB rebleeding, highlighting the need to consider endoscopic features in EVB patients to optimize risk assessment and management^16^.

There are several strengths of our study. First, the model incorporated key endoscopic findings, Radiological features and comorbidity information, factors often overlooked in previous studies, thereby leveraging a multidimensional set of clinical data to enhance predictive accuracy while balancing simplicity and predictive power with only eight key clinical features. Second, after comparing multiple ML models, the best-performing model was selected to ensure the final model achieved the highest predictive accuracy and robustness. Third, the model underwent independent validation using a prospective cohort, further demonstrating its generalizability across different datasets. Finally, we developed an online prediction platform to help clinicians quickly assess rebleeding risk, thereby facilitating the identification of high-risk patients and enabling more intensive endoscopic surveillance to mitigate rebleeding risk.

However, this study has certain limitations. First, the model was constructed with retrospective data, which may introduce inherent biases. Nonetheless, the strong performance of the XGBoost model in the prospective validation cohort supports its reliability. Second, while multiple clinical features were analyzed, some potential rebleeding risk factors, such as spleen stiffness and blood electrolyte levels, may have been overlooked. Therefore, additional prospective studies are needed to investigate these risk factors further. Third, due to limited case numbers, the study’s findings have yet to be validated in patient populations with non-alcoholic fatty liver cirrhosis, drug-induced cirrhosis, or schistosomal cirrhosis. To address these limitations and translate the model into practice, future large-sample, multi-center prospective studies should assess the clinical utility of implementing risk-stratified follow-up strategies based on this model. Additionally, incorporating new clinical features and other potential biomarkers may further enhance predictive accuracy and address some of the limitations identified in this study.

## Conclusion

We developed and validated an XGBoost model based on eight easily accessible clinical features to predict the 1-year rebleeding risk in patients with cirrhosis and EVB following EVL treatment. Among various ML models, the XGBoost model demonstrated the best predictive performance. Additionally, we developed an online risk prediction platform based on this model, providing clinicians with a rapid, individualized tool for rebleeding risk assessment, thereby supporting more rational, risk-stratified follow-up planning.

## Electronic Supplementary Material

Below is the link to the electronic supplementary material.

Supplementary Material 1

## Data Availability

Data supporting the findings of this study are available from the corresponding author upon reasonable request.

## Abbreviations

EVB: Esophageal variceal bleeding
EVL: Endoscopic variceal ligation
ML: Machine learning
TIPS: Transjugular intrahepatic portosystemic shunt
RFE: Recursive feature elimination
DT: Decision tree
RF: Random forest
KNN: k-nearest neighbors
LR: Logistic regression
XGBoost: Extreme gradient boosting
MLP: Multilayer perceptron
SVM: Support vector machine
Light GBM: Light gradient boosting machine
AUC: Area under the receiver operating characteristic curve
PR: Precision-recall
DCA: Decision curve analysis
NSBB: Non-selective beta-blockers
SHAP: Shapley additive explanations
ALB: Albumin
ALT: Alanine aminotransferase
AST: Aspartate aminotransferase
UA: Uric acid
PVT: Portal vein thrombosis
Pvv: Portal vein flow velocity
