# Supplementary material for: Machine learning model for predicting rebleeding risk after endoscopic variceal ligation in esophageal variceal bleeding
Source: Sci Rep. 2026 Jun 19;16:28160. doi: 10.1038/s41598-026-58733-2 (PMC13554231; doi:10.1038/s41598-026-58733-2)
Supplement: Supplementary file 1 [file 41598_2026_58733_MOESM1_ESM.docx]

**Machine Learning Model for Predicting Rebleeding Risk after Endoscopic Variceal Ligation in Esophageal Variceal bleeding**

**Authors**:

Junyi Zhan, Keqiang Lu, Jinzhong Yu, Jie Chen, Feifei Xing, Dexin Wang, Shihao Zhang, Mingyan Yang, Ping Liu, Jian Wang, Yongping Mu

**Correspondence to:**Yongping Mu, Ph.D. and M.D. Shuguang hospital affiliated to Shanghai University of Traditional Chinese Medicine, 528, Zhangheng Road, Pudong District, Shanghai 201203, China. E-mail: ypmu8888@126.com; Jian Wang, Ph.D. and M.D. Zhongshan hospital affiliated to Fudan University, 180, Fenglin Road, Xuhui District, Shanghai 200032, China. E-mail: wang.jian@zs-hospital.sh.cn.

Catalogs

**Supplementary figure 1**	**2**

**Supplementary figure 2**	**3**

**Supplementary figure 3**	**4**

**Supplementary figure 4**	**4**

**Supplementary table 1**	**5**

**Supplementary figure 1.** Feature selection using Recursive Feature Elimination.

**Supplementary figure 2.** Calibration curves of eight machine learning models in (A) the Training cohort and (B) the Validation cohort.

**Abbreviations:** dt: decision tree; rf: random forest; xgboost: extreme gradient boosting; svm: support vector machine; mlp: multilayer perceptron; lightgbm: light gradient boosting machine; knn: k-nearest neighbors.

**Supplementary figure 3.** Decision Curve Analysis curves of XG Boost model predicting EVB rebleeding in (A) the Training cohort and (B) the Validation cohort.

**Supplementary figure 4.** The SHAP dependence plots for the eight clinical predictors.

**Abbreviations:** ALT: alanine aminotransferase; AST: aspartate aminotransferase; ALB: albumin; HB: hemoglobin; PVT: portal vein thrombosis.

Supplementary table 1. Comparative baseline characteristics of the non-rebleeding and rebleeding cohorts.

| **Characteristic** | **Non-Rebleeding**  **cohort (n=378)** | **Rebleeding**  **cohort (n=114)** | **Missing data (%)** | ***P*-Value** |
| --- | --- | --- | --- | --- |
| **Demographics characteristics** |  |  |  |  |
| Age (yr) | 58.11±12.26 | 59.92±12.51 | 0 | 0.17 |
| Sex (%) |  |  | 0 | 0.54 |
| Female | 145 (38.36) | 48 (42.11) |  |  |
| Male | 233 (61.64) | 66 (57.89) |  |  |
| Smoking history (%) | 57 (15.16) | 26 (22.81) | 0.4 | 0.08 |
| Drinking history (%) | 301 (80.05) | 77 (67.54) | 0.4 | 0.01 |
| Surroundings (%) |  |  | 0 | 0.89 |
| Rural | 117 (30.95) | 33 (28.95) |  |  |
| Suburban | 67 (17.72) | 22 (19.30) |  |  |
| Urban | 194 (51.32) | 59 (51.75) |  |  |
| Symptom (%) |  |  | 0 | 0.01 |
| Melena or hematochezia | 143 (37.83) | 27 (23.68) |  |  |
| Hematemesis | 235 (62.17) | 87 (76.32) |  |  |
| Etiology (%) |  |  | 0 | 0.97 |
| Chronic HBV infection | 175 (46.30) | 51 (44.74) |  |  |
| Autoimmune liver disease | 69 (18.25) | 21 (18.42) |  |  |
| Alcohol-related | 56 (14.81) | 19 (16.67) |  |  |
| Other | 78 (20.63) | 23 (20.18) |  |  |
| Course of cirrhosis (year) | 2 (0.42-6) | 3 (0.9-7) | 1.6 | 0.02 |
| Family history of cirrhosis (%) | 38 (10.08) | 20 (17.54) | 0.2 | 0.05 |
| **Endoscopic findings** |  |  |  |  |
| Received emergency endoscopic hemostasis (%) | 25 (6.61) | 21 (18.42) | 0 | <0.01 |
| Types of varices (%) |  |  | 0 | 1 |
| EV | 159 (42.06) | 48 (42.11) |  |  |
| EV combined with GV | 219 (57.94) | 66 (57.89) |  |  |
| Red color sign (%) | 356 (94.18) | 110 (96.49) | 0 | 0.47 |
| Bleeding signs (%) | 42 (11.11) | 41 (35.96) | 0 | <0.01 |
| Form of varices (%) |  |  | 0 | 0.14 |
| F1 | 71 (18.78) | 14 (12.28) |  |  |
| F2-F3 | 307 (81.22) | 100 (87.72) |  |  |
| Location of varices (%) |  |  | 0.2 | 0.07 |
| Low to middle | 326 (86.24) | 89 (78.76) |  |  |
| High | 52 (13.76) | 24 (21.24) |  |  |
| Numbers of varices | 4 (4-4) | 4 (4-4) | 0 | 0.33 |
| Diameter of varices (cm) | 0.8 (0.8-1) | 1 (0.8-1.2) | 0.4 | <0.01 |
| Number of EVL (times) | 2 (1-3) | 2 (1-2.75) | 0 | <0.01 |
| Received sclerotherapy injections (%) | 168 (44.44) | 57 (50) | 0 | 0.35 |
| Received tissue glue injections (%) | 153 (40.48) | 51 (44.74) | 0 | 0.48 |
| Treatment failure (%) | 1 (0.26) | 3 (2.63) | 0 | 0.06 |
| **Comorbidities and complications** |  |  |  |  |
| Ascites (%) |  |  | 0 | <0.01 |
| None | 164 (43.39) | 5 (4.39) |  |  |
| Mild | 132 (34.92) | 64 (56.14) |  |  |
| Moderate and severe | 82 (21.69) | 45 (39.47) |  |  |
| Encephalopathy (%) | 9 (2.38) | 7 (6.14) | 0 | 0.09 |
| Diabetes (%) | 82 (21.75) | 20 (17.54) | 0.2 | 0.40 |
| Hypertension (%) | 81 (21.49) | 23 (20.18) | 0.2 | 0.87 |
| Hemorrhagic shock (%) | 3 (0.80) | 8 (7.02) | 0.2 | <0.01 |
| Heart disease (%) | 17 (4.51) | 8 (7.02) | 0.2 | 0.41 |
| **Laboratory parameters** |  |  |  |  |
| Total bilirubin (μmol/L) | 18.1 (13.07-26.58) | 21 (15.1-32.95) | 1.8 | <0.01 |
| Alanine aminotransferase (U/L) | 23 (16-32) | 25 (16-43.35) | 2.6 | 0.02 |
| Aspartate aminotransferase (U/L) | 31 (24-41) | 39 (24-55) | 2.4 | <0.01 |
| Alkaline phosphatase (U/L) | 85 (66-113) | 90 (67-135.75) | 3.3 | 0.25 |
| Albumin (g/L) | 34.21±5.67 | 29.12±6.15 | 2 | <0.01 |
| Globulin (g/L) | 28.55 (24.85-33) | 29 (25-33.5) | 3.5 | 0.64 |
| Blood urea nitrogen (mmol/L) | 5.3 (4.1-6.5) | 6.2 (4.77-9.03) | 4.5 | <0.01 |
| Creatinine (μmoI/L) | 67 (57-77.7) | 68.88 (53.75-85) | 3 | 0.43 |
| Uric acid (μmoI/L) | 288 (240.75-357.33) | 337.5 (239-421.5) | 2.8 | 0.03 |
| Total cholesterol (mmol/L) | 3.29 (2.65-4.04) | 2.89 (2.46-3.48) | 11.8 | <0.01 |
| White blood cell (×10^9^ /L) | 2.76 (1.98-4.21) | 3.78 (2.3-5.81) | 3.5 | <0.01 |
| Neutrophil (×10^9^ /L) | 1.7 (1.2-12.87) | 2.36 (1.3-4.21) | 3.5 | <0.01 |
| Lymphocyte (×10^9^ /L) | 0.7 (0.5-0.98) | 0.7 (0.5-1.08) | 4.3 | 0.23 |
| Red blood cell (×10^9^ /L) | 3.17±0.74 | 2.85±0.79 | 3 | <0.01 |
| Hemoglobin (g/L) | 91.03±23.56 | 80.02±24.27 | 1.6 | <0.01 |
| Platelet count (×10^9^ /L) | 66 (45-92) | 73 (50-96.75) | 1.8 | 0.31 |
| Prothrombin time (s) | 14.5 (13.57-15.7) | 14.95 (13.72-16.8) | 2 | 0.02 |
| INR (unit) | 1.26 (1.16-1.35) | 1.3 (1.18-1.44) | 2.4 | 0.01 |
| D-dipolymer (ug/ml) | 1.25 (0.5-2.79) | 1.93 (0.87-4.35) | 4.9 | <0.01 |
| Thrombin time (s) | 17.7 (16.7-18.8) | 17.65 (16.38-19.25) | 4.7 | 0.90 |
| Fibrinogen (g/L) | 195.26±68.16 | 200.55±101.38 | 4.1 | 0.53 |
| CTP class, n (%) |  |  | 0 | <0.01 |
| A (5-6) | 181 (47.88) | 14 (12.28) |  |  |
| B (7-9) | 183 (48.41) | 82 (71.93) |  |  |
| C (10-13) | 14 (3.70) | 18 (15.79) |  |  |
| **Radiological features** |  |  |  |  |
| Portal vein thrombosis (%) | 65 (17.20) | 58 (52.73) | 0.8 | <0.01 |
| Splenic vein diameter (mm) | 9 (7-11) | 10 (8.3-12) | 5.1 | 0.01 |
| Inner diameter of portal vein trunk (mm) | 12.6 (11-14) | 13 (12-14.25) | 4.1 | 0.01 |
| Portal vein flow velocity (cm/s) | 19.3 (16-23) | 20 (13-24) | 3.9 | 0.36 |
| **Medication usage** |  |  |  |  |
| Use of NSBB (%) | 258 (68.25) | 70 (61.40) | 0 | 0.21 |
| Use of PPI (%) | 356 (94.18) | 108 (94.74) | 0 | 1 |
| Use of antibiotics (%) | 259 (68.52) | 85 (74.56) | 0 | 0.26 |

**Abbreviations:** EV: esophageal varices; GV: gastric varices; EVL: endoscopic variceal ligation; INR: international normalized ratio; CTP class: Child-Turcotte-Pugh class; NSBB: non-selective beta-blockers; PPI: proton pump inhibitor.
